# 3D-conformal Accelerated Partial Breast Irradiation treatment planning: the value of surgical clips in the delineation of the lumpectomy cavity

**DOI:** 10.1186/1748-717X-4-70

**Published:** 2009-12-31

**Authors:** Maia Dzhugashvili, Elodie Tournay, Charlotte Pichenot, Ariane Dunant, Eduardo Pessoa, Adel Khallel, Sébastien Gouy, Catherine Uzan, Jean-Rémy Garbay, Françoise Rimareix, Marc Spielmann, Philippe Vielh, Hugo Marsiglia, Céline Bourgier

**Affiliations:** 1 Department of Radiation Oncology, Institut Gustave Roussy, Villejuif, France; 2 Department of Physics, Institut Gustave Roussy, Villejuif, France; 3 Biostatistics, and Epidemiology Unit, Institut Gustave Roussy, Villejuif, France; 4 Department of Breast Surgery, Institut Gustave Roussy, Villejuif, France; 5 Department of Breast Oncology, Institut Gustave Roussy, Villejuif, France; 6 Department of Pathology, Institut Gustave Roussy, Villejuif, France; 7 University of Florence, Italy

## Abstract

**Background:**

Accurate localisation of the lumpectomy cavity (LC) volume is one of the most critical points in 3D-conformal Partial breast irradiation (3D-APBI) treatment planning because the irradiated volume is restricted to a small breast volume. Here, we studied the role of the placement of surgical clips at the 4 cardinal points of the lumpectomy cavity in target delineation.

**Methods:**

Forty CT-based 3D-APBI plans were retrieved on which a total of 4 radiation oncologists, two trainee and two experienced physicians, outlined the lumpectomy cavity. The inter-observer variability of LC contouring was assessed when the CTV was defined as the delineation that encompassed both surgical clips and remodelled breast tissue.

**Results:**

The conformity index of tumour bed delineation was significantly improved by the placement of surgical clips within the LC (median at 0.65). Furthermore, a better conformity index of LC was observed according to the experience of the physicians (median CI = 0.55 for trainee physicians *vs *0.65 for experienced physicians).

**Conclusions:**

The placement of surgical clips improved the accuracy of lumpectomy cavity delineation in 3D-APBI. However, a learning curve is needed to improve the conformity index of the lumpectomy cavity.

## Background

Accelerated Partial Breast Irradiation (APBI) is still under investigation to demonstrate equivalence to whole breast irradiation in terms of local control. Among the different APBI techniques (invasive or non-invasive), 3D-conformal APBI is widely used given its accessibility in radiotherapy centres [1]. However, several issues related to this technique still warrant investigation: e.g. the identification and contouring of the lumpectomy cavity (LC), the patient's set-up and optimal dose determination. The definition of the lumpectomy cavity is an essential part of 3D-conformal APBI treatment planning as the irradiation is confined to a limited volume of breast tissue adjacent to the lumpectomy cavity. Unlike intra-operative partial breast irradiation, LC determination is critical as treatment delivery is delayed after breast surgery. In 3D-APBI, the GTV (Gross Tumour Volume) and CTV (Clinical Target Volume) are generally defined as the contouring of a seroma within the lumpectomy cavity, expanded by a 1 cm margin [2,3]. However, the delineation of the seroma could vary among different observers and even among experienced ones[4].

In France, breast tissues are usually remodelled after lumpectomy. Consequently, none or a few lumpectomy cavities with seroma are visible. In such cases, it is more difficult to delineate the LC. In order to better visualize the lumpectomy cavity after breast tissue remodelling at the Institut Gustave Roussy (IGR), the surgical process in breast cancer consists in systematically placing 4 surgical clips within the lumpectomy cavity, in order to locate the LC in the ongoing APBI trial [5]. In the IGR APBI trial, target delineation consists in outlining the surgical clips and the visible lumpectomy cavity (surgically remodelled breast tissue) instead of delineating seroma; and the CTV is considered equivalent to the GTV. Then the PTV is uniformly expanded by 1.5 to 2.0 cm around the CTV.

In a previous study [5], we showed that surgical clips were needed to locate the lumpectomy cavity. In the present study, we investigated whether surgical clips would improve the conformity index when a seroma is less or not visible.

## Methods and materials

### Breast Surgery Procedures and Treatment Planning

**Figure 1:**
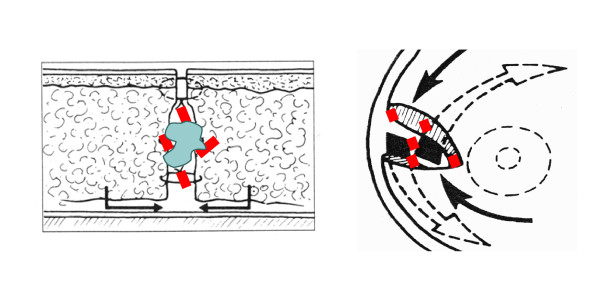
**Surgical procedure of clip placement**.

From January 2008 to April 2008, 40 patients underwent breast-conserving surgery which included a lumpectomy, placement of surgical clips at the four cardinal points of the LC and then breast tissue remodelling and an axillary node biopsy or dissection. The breast tissue remodelling technique consisted in mobilizing glandular tissues adjacent to the tumour bed after wide cutaneo-glandular detachment, and suturing them together. This procedure implies that none or few lumpectomy cavities with seroma are visible. When breast tissue remodelling is performed without surgical clip placement, visualization of the lumpectomy cavity is really difficult and this jeopardizes adequate localisation of the tumour bed, which is essential for APBI [5]. In the present study, 4 experienced breast surgeons in a single Institution performed breast lumpectomies and placed clips within the tumour bed according to the surgical placement procedure, i.e. 4 clips were placed at the upper, inner, outer and lower surgical margins of the tumour bed (Figure 1).

All patients had a computed tomography (CT)-based simulation for postoperative breast irradiation in the treatment position, *i.e*. in the supine position on the Med Tec inclined breast board with arms up (Model MT-350-N). The clinical breast borders, the LC scar and the post-surgical indurations were outlined with radio-opaque wires. The CT images extended approximately from the upper-clavicle to the upper abdomen in 2 or 4-mm thick slices (Siemens SOMATOM Sensation Open/Siemens Navigator/SOMARIS/5 Syngo).

Two experienced radiation oncologists specialized in breast cancer radiotherapy (Experts E1 and E2) and 2 trainee radiation oncologists (Juniors J1 and J2) blindly delineated 40 consecutive lumpectomy cavities without reviewing the contours of the other observers. Expertise was defined as the delineation of more than 400 breast CT scans per year for at least 2 years, and trainee physicians either had no prior experience in outlining breast CT scans or had less than 6 months experience. All volume delineation was performed on axial slices and on sagittal and/or coronal views if needed, on the Dosisoft/Isogray virtual simulation software (version 4.0.05 gL). The 4 trainee and expert radiation oncologists were allowed to choose how they wished to outline the target volume (the CTV was considered equivalent to the GTV). If they felt they should only include the "seroma", they exclusively outlined the "seroma". If on the other hand they felt they should outline both the seroma and the remodelled breast tissue, they outlined both. In all cases, medical and surgical files were available if needed.

In this study, we exclusively assessed inter-observer variability of LC delineation, focusing (i) on the entire CTV (CTV = surgical clips + remodelled breast tissue); (ii) only on the study of the CTV when it was restricted to the CT slices which contained clips; and finally (iii) on the remodelled breast tissue.

### Analysis of contoured volumes

Inter-observer variability was quantified according to the 3 assays described by Landis *et al*.: (i) Variability of CTV volume (cm^3^); (ii) The Percent Volume Overlap; (iii) and the Centre Of the Mass assay (COM) [4]. All of these parameters were calculated by the virtual simulation software. Then, inter-observer variability was assessed (1) using the distance between each COM that was calculated for each pairwise volume comparison; (2) using the number of slices including a recorded contour; (3) and using the percent volume overlap obtained between pairwise volumes (Microsoft/Excel 2003 software). Finally, interobserver variability was evaluated using the conformity index (CI) of two delineated structures (V1 and V2). The CI was defined as the ratio of the overlapping volume V1UV2 and the encompassing total delineated volume V1UV2.

### Statistical methods

Normality was tested using the Kolmogorov Smirnov test. As variables did not follow a normal distribution, data were summarized with medians (minimum-maximum). Comparisons within studies 1 and 2 or between readers were made using the Wilcoxon matched-pair test. All tests were two-sided, and p-values below 0.01 were considered to denote statistical significance because of multiple tests. Statistical analyses were performed using SAS software, version 9.1 (SAS Institute, Cary, NC).

## Results

### Patient demographics

Median age was 60 years (range, 44-94 years). Median time from surgery to CT scan simulation was 51 days (range, 14-252). The median surgical lumpectomy volume based on histological reports was 76 cm^3 ^(range, 13-364 cm^3^). The median tumour size was 12 mm (range, 2-35 mm) and most of the patients had a pT1N0 breast cancer (73%). Chemotherapy was administered to 13 patients before breast radiotherapy (33%) (Table 1).

**Table 1:** Patient demographics and clinical data.

N = 40	Median	Min - max	N (%)
Age (years old)	60	44 - 94	

Tumour size (mm)	12	2 - 35	

Histological tumour volume (cm^3^)	76	13 - 364	

Time from surgery to radiotherapy (days)	51	14 - 252	

Adjuvant chemotherapy (number of patients)			13 (33%)

pTis pN0			1 (2.5%)

pT1 pN0-1			33 (82.5%)

pT2 pN0-1			6 (15%)

### CTV Volume (Table 2 and 3)

**Table 2:** Discrepancies in lumpectomy cavity volumes between the 4 radiation oncologists (Juniors J1 and J2; Experts E1 and E2).

N = 40	Volume of lumpectomy cavity (cm^3^)		Volume of lumpectomy cavity (cm^3^) when the assessment was restricted to CT slices containing surgical clips		p
	**Median**	**Min - max**	**Median**	**Min - max**	

J1	14	5 - 53	15	6 - 53	0.98

J2	16	5 - 61	16	5 - 60	0.39

E1	13	5 - 48	12	5 - 46	p < 0.0001

E2	11	5 - 96	11	4 - 50	p < 0.0001

**Table 3:** Discrepancies in lumpectomy cavity (LC) volumes; in conformity index and in distances between centres of gravity of each LC volume when the study was restricted to CT slices containing surgical clips.

		Volume of lumpectomy cavity - cm^3 ^(n = 40)	P*	Conformity index	Distance between gravity centres (mm)
ΔJ1-J2	Med (min; max)	- 0.9 (-17.9; 13.1)	0.02	0.64 (0.36; 0.80)	2.2 (0.0; 11.9)

ΔJ1-E1	Med (min; max)	1.5 (-4.5; 17.7)	0.007	0.65 (0.13; 0.82)	1.8 (0.5; 26.3)

ΔJ1-E2	Med (min; max)	3.5 (-20.9; 24.5)	< 0.0001	0.57 (0.09; 0.77)	2.4 (0.6; 30.9)

ΔJ2-E1	Med (min; max)	3.5 (-4.9; 22.8)	< 0.0001	0.57 (0.24; 0.86)	2.4 (0.7; 20.4)

ΔJ2-E2	Med (min; max)	4.2 (-7.5; 27.1)	< 0.0001	0.55 (0.07; 0.88)	3.1 (1.3; 24.7)

ΔE1-E2	Med (min; max)	1.6 (-27.4; 10.2)	< 0.0002	0.62 (0.38; 0.73)	2.51 (0.2; 10.0)

* p value for testing equality of volumes between physicians ( < 0.01 is significant).

ΔJ1-J2 = between J1 and J2. ΔJ1-E1 = between J1 and E1. ΔJ1-E2 = between J1 and E2. ΔJ2-E1 = between J2 and E1. ΔJ2-E1 = between J2 and E2. ΔE1-E2 = between E1 and E2.

Contouring by juniors was much larger, even though these volumes were restricted exclusively to slices containing clips (excess volume of 2 cm^3^, p = 0.03 and 0.002). Indeed, remodelled breast tissue was not outlined by the juniors but was almost exclusively outlined within the target volumes by both experienced radiation oncologists (p < 0.0001) (Table 2). In the case of experts, the volumes were more extensive in terms of the number of CT slices, because they included remodelled breast tissue. Yet in spite of incorporating remodelled breast tissue, their volumes were still smaller than those of trainees. Discrepancies were observed more particularly between experts than between juniors (p = 0.004). They mostly concerned the remodelled breast tissue, and only to a small extent the part containing the surgical clips.

### Number of CT slices used to delineate CTV

Experts outlined their CTV volume on more CT slices (median of 11 CT slices) than junior radiation oncologists (median of 9 CT slices). Besides a greater number of CT slices, the upper and lower limits of the lumpectomy cavity were not always concordant between the experienced radiation oncologists (p < 0.0001) whereas they were concordant when delineated by juniors as they only used CT slices which contained surgical clips (p = 0.19). In conclusion, the experienced radiation oncologists outlined the lumpectomy cavity on CT slices which contained both surgical clips and remodelled breast tissue whereas juniors restrained their CTV delineation to CT slices which exclusively contained surgical clips.

### Conformity index (CI) of CTV delineation (Figure 2a and 2b)

**Figure 2:**
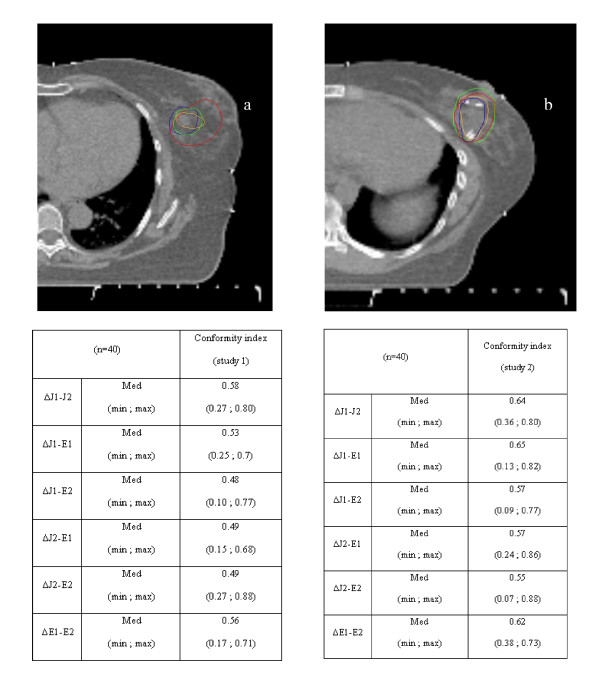
**Surgical clips improved the Conformity Index**. Figure 2a, CI without clips; Figure 2b, CI with clips. In each figure, lumpectomy cavity delineation according to each physician (green: J1; red: J2; orange: E1 and blue: E2).

when the entire CTV was assessed, the CI was relatively low, ranging from 0.48 (range = 0.10 - 0.77) to 0.58 (range = 0.27 - 0.8). The CI was more concordant between juniors (CI = 0.58) than between experts (CI = 0.56), but was clearly lower for juniors with a CI at 0.48 (range = 0.1-0.77) compared to experts with a CI at 0.53 (range = 0.25-0.73). When the assessment of the CTV was restricted to CT slices containing surgical clips (Table 3), the CI was improved, ranging from 0.55 to 0.65. The CI was more concordant between juniors with a CI at 0.64 (range = 0.36 - 0.8) than between experts with a CI at 0.62 (range = 0.38 - 0.73). Once again, the CI of the CTV delineated by junior radiation oncologists was clearly lower with a median CI at 0.55 (range = 0.07-0.88) than the CTV delineated by experienced radiation oncologists with a median at 0.65 (range = 0.13-0.82). Finally, a higher concordance of CTV delineation was related to experience and to the presence of surgical clips even though, given the limited number of physicians involved in this study, the results should exclusively be interpreted at the individual rather than at the collective level.

### Centre of the Mass (COM) of CTV (Table 4)

**Table 4:** Discrepancies in distances inter-Com (Centre Of the Mass of the target volume) when the CTV was entirely assessed (study 1) and when the study was restricted to CT slices containing surgical clips (study 2).

	Study 1 Distance inter-Com (mm)		Study 2 Distance inter-Com (mm)	
	
	Median	Min - max	Median	Min - max
ΔJ1-J2	3.4	0 - 11.9	2.2	0 - 11.9

ΔJ1-E1	2.6	0.9 - 34.2	1.8	0.5 - 26.3

ΔJ1-E2	3.3	1.1 - 36.5	2.4	0.6 - 30.9

ΔJ2-E1	3.3	0.9 - 24.4	2.4	0.7 - 20.4

ΔJ2-E2	3.5	1.3 - 26.2	3.1	1.3 - 24.7

ΔE1-E2	3.1	0.2 - 24.3	2.5	0.2 - 10.0

ΔJ1-J2 = between J1 and J2. ΔJ1-E1 = between J1 and E1. ΔJ1-E2 = between J1 and E2. ΔJ2-E1 = between J2 and E1. ΔJ2-E1 = between J2 and E2. ΔE1-E2 = between E1 and E2.

The Distance inter-COM (DICOM) between the different observers ranged from +2.6 mm to +3.5 mm. The DICOM was higher between juniors (DICOM = +3.4 mm) than between experts (DICOM = +3.1 mm). When we restricted the study of CTV delineation to the CT slices containing surgical clips, the distances between the centres of gravity of each LC volume were smaller, ranging from +1.8 mm to +3.1 mm between the different observers.

## Discussion

This study showed that the placement of surgical clips improved the conformity index with less inter-observer variability in the definition of the lumpectomy cavity after breast-conserving surgery. Different surrogates have been used to define the lumpectomy cavity for the tumour boost: the scar on breast [6], placement of surgical clips [7-9] or imaging by ultrasound or CT scan [10,11]. Regarding surgical clips, their use could vary in terms of their number [from one to multiple clips] [12] and in terms of their placement [prepectoral or within the LC]. In 3D-conformal APBI, no consensus has been reached concerning surgical clip placement. In the NSABP B39/RTOG 0413 trial, the definition of the target volume is based either on surgical clips, if present or on seroma if clearly visible. Then the CTV is expanded by 15 mm around the GTV [13]. In the APBI-MGH trial, the GTV and the CTV are considered as equivalent [3]. Surgical clips were not required in either protocol whereas clip placement is mandatory in the ongoing IGR APBI trial. Nevertheless, the target volume in our study was in the same range as those described in the literature [2,14-16] (Table 5) even though remodelled breast tissue was included in CTV delineation.

**Table 5:** Lumpectomy cavity (LC) volumes in APBI trials

		Median volume of lumpectomy cavity (cc)	Min - max (cc)
Berrang et al [13]	Seroma visible on CT-scan	31.8	7.3 - 131.9
	
	Seroma visible on 3D-US	23.2	5 - 93.6

Ford et al [14]	LC on CT scan	15.4	5.2 - 133.5
	
	LC on PET-CT	32.8	7 - 199.4

Vicini et al [1]	LC	12	5 - 65

Formenti et al [12]	LC	34	7 - 379

IGR study	LC by juniors	14-16	5 - 61
	
	LC by experts	11-13	5 - 96

The present study showed that surgical clips placed at the 4 cardinal points of the lumpectomy cavity improved the conformity index between the different observers, from 49% to 65%. However, defining the lumpectomy cavity using surgical clips within the lumpectomy cavity still seems insufficient for adequately defining the GTV in 3D-conformal APBI. Indeed, some issues regarding LC contouring still need to be more clearly characterized such as the need to outline breast tissue changes, the expansion of seroma margins etc. Thus, other imaging techniques are needed, such as either ultrasound, positron emission tomography/CT (PET-CT) that identifies inflammatory tissue remodelling or magnetic resonance imaging (MRI). Recently, Berrang *et al. *reported on the usefulness of combining 3D-breast ultrasound (3D-US) with the CT scan in treatment planning for APBI. Indeed, they showed that less inter-observer variability was seen when contouring seroma with 3D-US, with smaller seroma volumes when they were outlined using 3D-US [15]. However, 3D-US has never been evaluated in cases of remodelled breast tissue alone, the most frequent type of breast-conserving surgery in France, without the presence of seroma. PET-CT, another type of functional imaging has also been assessed. A recent study showed that the lumpectomy cavity was well visualized with PET-CT but contouring volumes were always larger than those outlined on CT scan [16]. However, PET-CT identified inflammatory tissue remodelling but not residual disease. Magnetic resonance imaging may be of interest for visualising and defining the postoperative lumpectomy cavity because of its superiority in terms of soft tissue contrast and better differentiation of normal tissue from the lumpectomy cavity. Recently, Kirby et al showed that MRI allowed a higher conformity index (CI at 0.89) when compared with CT scan. Even though MRI is of interest for CTV delineation, it is not readily accessible and when available, it is often performed in the prone position whereas treatments are performed in the supine position [17,18]. Further evaluations are needed to clearly define the role of either 3D-US or PET-CT or MRI in contouring LC.

Besides surgical clip placement, the present study also showed that the CI was higher with experience (CI for juniors at 0.55 and for experts at 0.65) suggesting the need for a learning curve. Indeed, a learning curve was highly recommended by Wong et al in the contouring of the lumpectomy cavity as the CTV observed among trainee radiation oncologists was always larger than those of trained physicians [19,20]. In our study, the LC volumes defined by the juniors were always larger than those of the experts because of their uncertainty regarding volume delineation. Juniors positioned their target volume close to surgical clips without including remodelled breast tissue. Although the experts outlined the LC volume with the remodelled breast and surgical clips, their LC volumes were still smaller than those of trainees.

## Conclusion

The definition of the lumpectomy cavity (GTV and CTV) is an essential part of 3D-APBI treatment planning. The placement of surgical clips at the 4 cardinal points of the lumpectomy cavity strongly improved the accuracy of target contouring. Therefore, the use of surgical clips in the delineation of LC in 3D-conformal APBI is required.

## List of abbreviations

LC: lumpectomy cavity; APBI: accelerated partial breast irradiation; CI: confidence interval; COM: Centre of the Mass assay; DICOM: Distance inter-COM; GTV: Gross Tumour Volume; CTV: Clinical Tumour Volume; PTV: Planning treatment volume; 3D-US: 3D-breast ultrasound; PET-CT: Positron emission tomography/CT; MRI: magnetic resonance imaging.

## Competing interests

The authors declare that they have no competing interests.

## Authors' contributions

MD: acquisition, analysis and interpretation of data; CP, ET, AD and HM: analysis and interpretation of data; EP, AK, SG, CU, JRG, FR, MS, PV: data acquisition; CB: conception, design. All the listed authors have been involved in drafting or in revising the manuscript. All authors read and approved the final manuscript.
